# Longevity of hybrid immunity against SARS-CoV-2 in adults vaccinated with an adenovirus-based COVID-19 vaccine

**DOI:** 10.1186/s12879-024-09891-z

**Published:** 2024-09-12

**Authors:** Memory Mvula, Fatima Mtonga, Jonathan Mandolo, Chisomo Jowati, Alice Kalirani, Precious Chigamba, Edwin Lisimba, Ndaona Mitole, Marah G. Chibwana, Kondwani C. Jambo

**Affiliations:** 1 Malawi-Liverpool-Wellcome Research Programme, Blantyre, Malawi; 2 University of Oxford, Oxford, England UK; 3 Liverpool School of Tropical Medicine, Liverpool, UK

**Keywords:** Hybrid immunity, Breakthrough infection, Longevity, SARS-CoV-2

## Abstract

**Background:**

Hybrid immunity provides better protection against COVID-19 than vaccination or prior natural infection alone. It induces high magnitude and broadly cross-reactive neutralising anti-Spike IgG antibodies. However, it is not clear how long these potent antibodies last, especially in the context of adenovirus-based COVID-19 vaccines.

**Methods:**

We conducted a longitudinal cohort study and enrolled 20 adults who had received an adenovirus-based COVID-19 vaccine before a laboratory-confirmed SARS-CoV-2 infection. We followed up the study participants for 390 days post the initial breakthrough infection. We assessed the longevity and cross-reactive breadth of serum antibodies against SARS-CoV-2 variants of concern (VOCs), including Omicron.

**Results:**

The binding anti-Spike IgG antibodies remained within the reported putative levels for at least 360 days and were cross-neutralising against Beta, Gamma, Delta, and Omicron. During the follow up period, a median of one SARS-CoV-2 re-infection event was observed across the cohort, but none resulted in severe COVID-19. Moreover, the re-exposure events were associated with augmented anti-Spike and anti-RBD IgG antibody titres.

**Conclusions:**

This study confirms that hybrid immunity provides durable broadly cross-reactive antibody immunity against SARS-CoV-2 variants of concern for at least a year (360 days), and that it is further augment by SARS-CoV-2 re-exposure.

## Introduction

Globally, most people have now acquired immunity against SARS-CoV-2 through vaccination or natural infection [1]. Studies have shown that anti-SARS-CoV-2 neutralising antibodies play an important role in protection against COVID-19 [2]. However, the potency of these antibodies tends to decline over time due to frequent mutations in the spike protein of SARS-CoV-2 [3]. This has led to the emergence of highly transmissible SARS-CoV-2 variants of concern, such as Omicron [4], which have caused a significant increase in breakthrough infections in COVID-19-vaccinated individuals [5].

Breakthrough infections occurring in vaccinated individuals induce hybrid immunity [6], which provides better protection than vaccination or prior natural infection alone [7, 8]. Hybrid immunity is associated with improved breadth and neutralising ability against multiple variants of concern [9–11]. It has been noted that the mRNA COVID-19 vaccines tend to induce greater protective antibodies than adenovirus-based vaccines [12–14], as a result the vaccine platform likely influences the quality and quantity of hybrid immunity. However, most of the available evidence of hybrid immunity has focused on immune responses in individuals who received 2 or 3 mRNA COVID-19 vaccines before followed by a SARS-CoV-2 infection [7, 8, 15]. Additionally, studies on hybrid immunity in adenovirus-based vaccinees have been limited to short-term follow-up of at most 182 days [16, 17]. As such, more information about the persistence of immunity conferred through hybrid immunity, especially using adenovirus-based COVID-19 vaccines, is needed to potentially predict immune protection against future SARS-CoV-2 variants and guide public health strategies.

To address this gap, we conducted a serological longitudinal study among COVID-19-vaccinated adults who had received an adenovirus-based COVID-19 vaccine followed by a SARS-CoV-2 breakthrough infection during the Delta and Omicron waves in Malawi.

## Methods

### Study setting and population

Using a longitudinal study design, individuals who had recovered from mild/moderate COVID-19 during the third and fourth epidemic waves (August 2021 and March 2022) were recruited in Blantyre City, Malawi. We used a convenience sampling approach, whereby the study was advertised electronically and by word of mouth, with support from the Blantyre District Health Office and Malawi-Liverpool-Wellcome Research Programme. Inclusion criteria for the study included adults aged between 18 and 65 years with a previous history of laboratory-confirmed SARS-CoV-2 infection. The exclusion criteria included withholding consent and having symptoms suggestive of COVID-19 at recruitment. Serum samples were collected at recruitment which was at least 30 days following the breakthrough infection and then every 30 days up to 390 days. We used electronic case report forms (eCRFs) to collect clinical history, vaccination history, and demographic data.

### Multiplex-based immunoassay for quantification of SARS-CoV-2 (S) spike, RBD and nucleocapsid IgG antibodies

A multiplex Meso-Discovery immunoassay (MSD) was used to measure SARS-CoV-2 IgG antibody titres against three viral proteins: Spike, Nucleocapsid and Receptor Binding Domain (RBD). The assay was performed using a 10-spot MULTI-SPOT 96-WELL plate that was pre-coated with eight different SARS-CoV-2 antigens, including RBD, Nucleocapsid, and Spike antigens for Wild type, Alpha, Beta, Gamma, Delta, and original Omicron (B.1.1.529), following the manufacturer’s instructions. In summary, the plates were blocked with MSD Blocker A, and reference standards, controls and samples diluted 1:5000 in diluent buffer were then added. After incubation, a detection antibody was added (MSD SULFO-TAG™ Anti-Human IgG Antibody), and then MSD GOLD™ Read Buffer B was added. The plates were then read using a MESO® QuickPlex SQ 120 MM Reader. As per the manufacturer's instructions for the MSD kit, only the wildtype spike, wildtype nucleocapsid, and wildtype RBD have measurements in both World Health Organization Binding Antibody Units (WHO BAU/ml) and Meso Scale Discovery Arbitrary Units (MSD AU/ml). However, the remaining variant of concern Spike antibody measurements only have MSD AU/ml units and are not yet calibrated to WHO BAU/ml units.

### ACE-2 inhibitory neutralisation assay

A quantitative COVID-19 ACE2 Neutralization MSD 10-spot MULTI-SPOT® 96-well plate was used to measure neutralisation capacity, following the manufacturer’s instructions. The plate was pre-coated with SARS-CoV-2 viral antigens and then blocked with MSD Blocker A for 30 minutes. Prepared reference standards and 1:5000 diluted samples in diluent buffer were added, followed by an incubation period of 2 hours. After that, ACE2 detection antibody (SULFO-TAG Human ACE2 Protein) was added and incubated for 60mins. Finally, MSD GOLD™ Read Buffer B was added and plates were read using a MESO® QuickPlex SQ 120 MM Reader.

### Statistical analysis

We performed statistical analyses and graphical presentations in R version 4.0.1 using RStudio (2022.07.1+554). The antibody binding and neutralisation activity data were log10 transformed. Wilcoxon rank sum test was used to compare log10 transformed data, with *the p*-value adjusted for multiple comparisons using the Bonferroni multiple comparisons test. For the sero-incidence data, Wilcoxon signed rank test was used. Effects were considered statistically significant when the *p*-value was less than 0.05.

## Results

### Participant demographics and clinical characteristics

A total of 20 recovered COVID-19 patients were recruited into the study (Table 1). The actual days for the different visit categories were day 60 [59(53-68)], day 90 [91(86-100)], day 120 [121(115-128)], day 150 [149(146-161)], day 180 [182(177-191)], day 210 [214(208-227)], day 240 [245(238-260)], day 270 [276(269-290)], day 300 [313(299-324)], day 330 [342(328-356)], day 360 [377(359-383)], and day 390 [404(386-414)]. All the study participants received at least a single dose of a COVID-19 vaccine (COVISHIELD™, *n*=19 or Janssen Ad26.COV2. S, *n=1*) as part of the routine vaccination programme. Of the 19 COVISHIELD™-vaccinated adults, 12 received two doses, and 7 received one dose. The median time from the first vaccine dose to laboratory-confirmed breakthrough infection was 138 days (IQR 112-253 days), and the median time since the last breakthrough infection at enrolment was 59 days (IQR 53-68). Of the 12 that received two doses of COVISHIELD™, 42% (*n*=5) experienced breakthrough infections after completing both doses and 58% (*n*=7) experienced breakthrough infections after the first dose. Although we did not perform SARS-CoV-2 genomic sequencing as part of the study, it’s worth noting that the recruitment period coincided with the third and fourth waves dominated by the Delta and Omicron variants, respectively [18]. Furthermore, none of the study participants experienced severe COVID-19 or hospitalisation during the follow-up period. Additionally, none of them reported any PCR-confirmed SARS-CoV-2 infection during the follow-up period.

**Table 1:** Participant demographics and site characteristics

	**Social demographics and clinical characteristics**		
**Characteristic**	**Overall, *N*=20**^**a**^	**Female, *N*=10**^**a**^	**Male, *N*=10**^**a**^
Age	30.0 (27.0, 36.3)	27.5 (26.3, 34.5)	31.0 (27.8, 37.5)
Vaccine type			
COVISHIELD™	19 (95%)	9 (47%)	10 (53%)
2 Doses	12 (63%)	6 (50%)	6 (50%)
1 Dose	7 (37%)	3 (43%)	4 (57%)
Janssen Ad26.COV2.S	1 (5%)	1 (10%)	0 (0%)

^a^n (%); Median (IQR)

### Kinetics and magnitude of serum anti-SARS-CoV-2-specific IgG antibodies

**Fig. 1:**
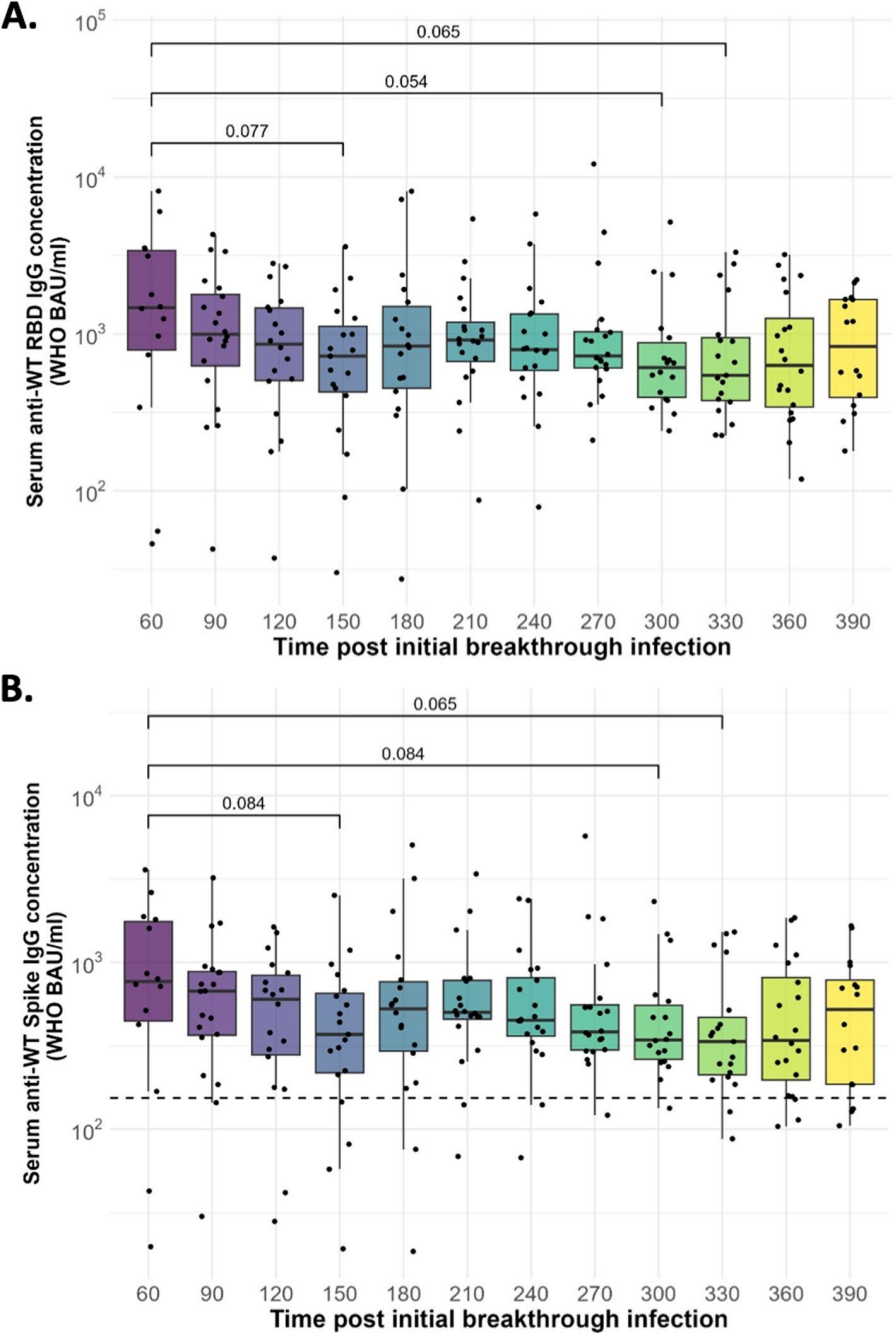
Serum IgG antibody kinetics. Box plots showing (**a**) anti-RBD IgG and (**b**) anti-Spike IgG antibody concentrations for *n*=20 individuals, post initial breakthrough infection. The dotted line represents reported putative protective anti-Spike IgG levels. The horizontal bars represent the median and interquartile range (IQR). Data was log 10 transformed and statistics were calculated using Wilcoxon rank sum test and *p* value adjusted for multiple comparisons using the Bonferroni multiple comparisons test. A *p*<0.05 was regarded as statistically significant. All visits were compared to day 60, and only *p* values <0.1 are shown on the graph. WT, Wild-type; RBD, Receptor Binding Domain; WHO BAU, World Health Organization Binding Antibody Units

**Fig. 2:**
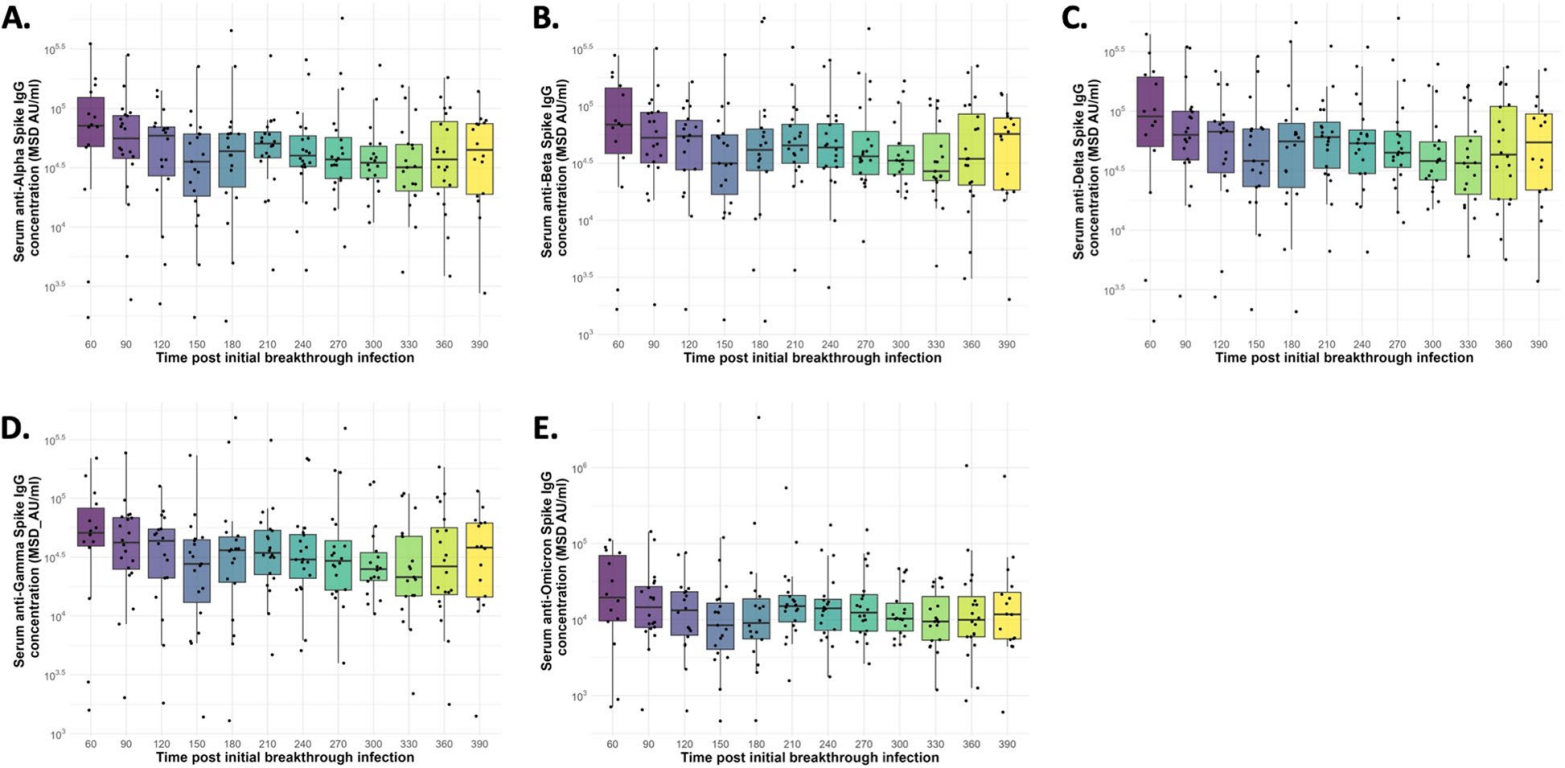
Anti-SARS-CoV-2 variants serum IgG antibody kinetics. Box plots showing (**a**) anti-Alpha Spike IgG, (**b**) anti-Beta Spike IgG, (**c**) anti-Gamma Spike IgG, (**d**) anti-Delta Spike IgG, and (**e**) anti-Omicron (B.1.1.529) Spike IgG antibody concentrations for *n*=20 individuals, post initial breakthrough infection. The horizontal bars represent the median and interquartile range (IQR). Data was log 10 transformed. MSD AU, Meso Scale Discovery Arbitrary Units

**Fig. 3:**
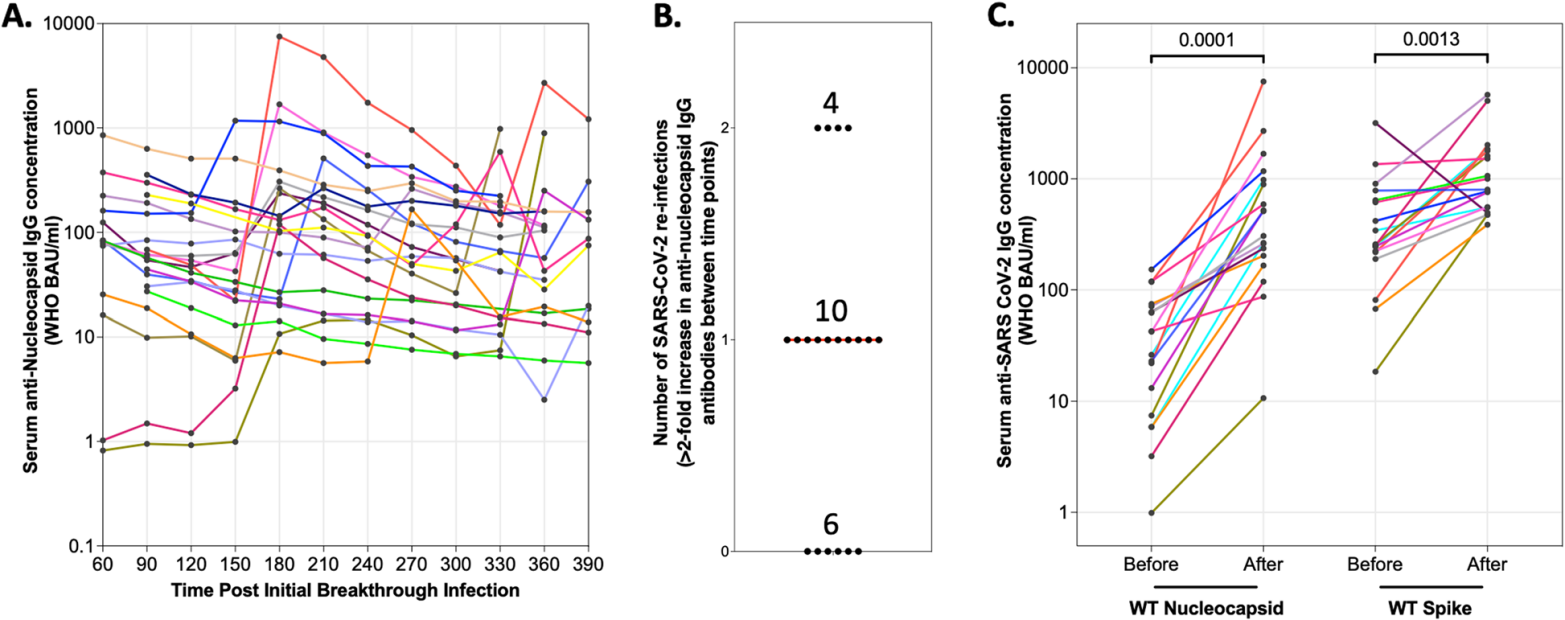
SARS-CoV-2 IgG Nucleocapsid antibody kinetics post initial breakthrough infection. **a** Anti-nucleocapsid concentrations overtime in serum samples after the first breakthrough infection (*n*=20). Data was log transformed. **b** Number of reinfections determined by a 2-fold increase in anti-Nucleocapsid IgG antibodies (*n*=20). **c** Corresponding increase in anti-Spike IgG antibodies in individuals with 2-fold increase in anti-Nucleocapsid IgG antibodies (*n*=14). Data was log 10 transformed and statistics were calculated using Wilcoxon signed rank test. WHO BAU, World Health Organization Binding Antibody Units

We assessed the kinetics of binding anti-SARS-CoV-2 IgG antibodies by measuring the levels of serum anti-RBD, anti-Spike, and anti-nucleocapsid-specific IgG antibodies throughout the follow-up period. The levels of anti-RBD and anti-Spike-specific IgG antibodies trended towards a decrease over time but remained within the levels associated with protection against symptomatic infection with wild-type, alpha, and delta variant SARS-CoV-2 virus [19] until at least one year post-hybrid immunity (Fig. 1a-b). The trend was also similar for anti-Spike IgG antibodies against the SARS-CoV-2 variants of concern (Fig. 2a-e). A notable waning of anti-nucleocapsid IgG antibodies was observed in the first 150 days post-initial breakthrough infection, followed by a rise in the antibody levels, which gradually waned thereafter (Fig. 3a). Furthermore, individual kinetics of anti-nucleocapsid IgG antibodies were used to calculate sero-incidence indicating a median of 1 peak per individual during the study follow-up period (1 peak [95% CI, 0-1]) (Fig. 3b), which suggested SARS-CoV-2 re-infection. As previously reported [20], the re-infection events were determined by a 2-fold increase in anti-nucleocapsid IgG antibodies between time points. Moreover, all individuals with a 2-fold in anti-nucleocapsid IgG antibodies between time points had a corresponding anti-Spike IgG antibody rise, except one individual (Fig. 3c). Overall, this indicates that binding IgG antibodies in vaccinated adults induced following breakthrough infection are durable and likely maintained at protective levels through non-severe SARS-CoV-2 re-infections.

### Neutralisation breadth of serum anti-SARS-CoV-2-specific antibodies over time

**Fig. 4:**
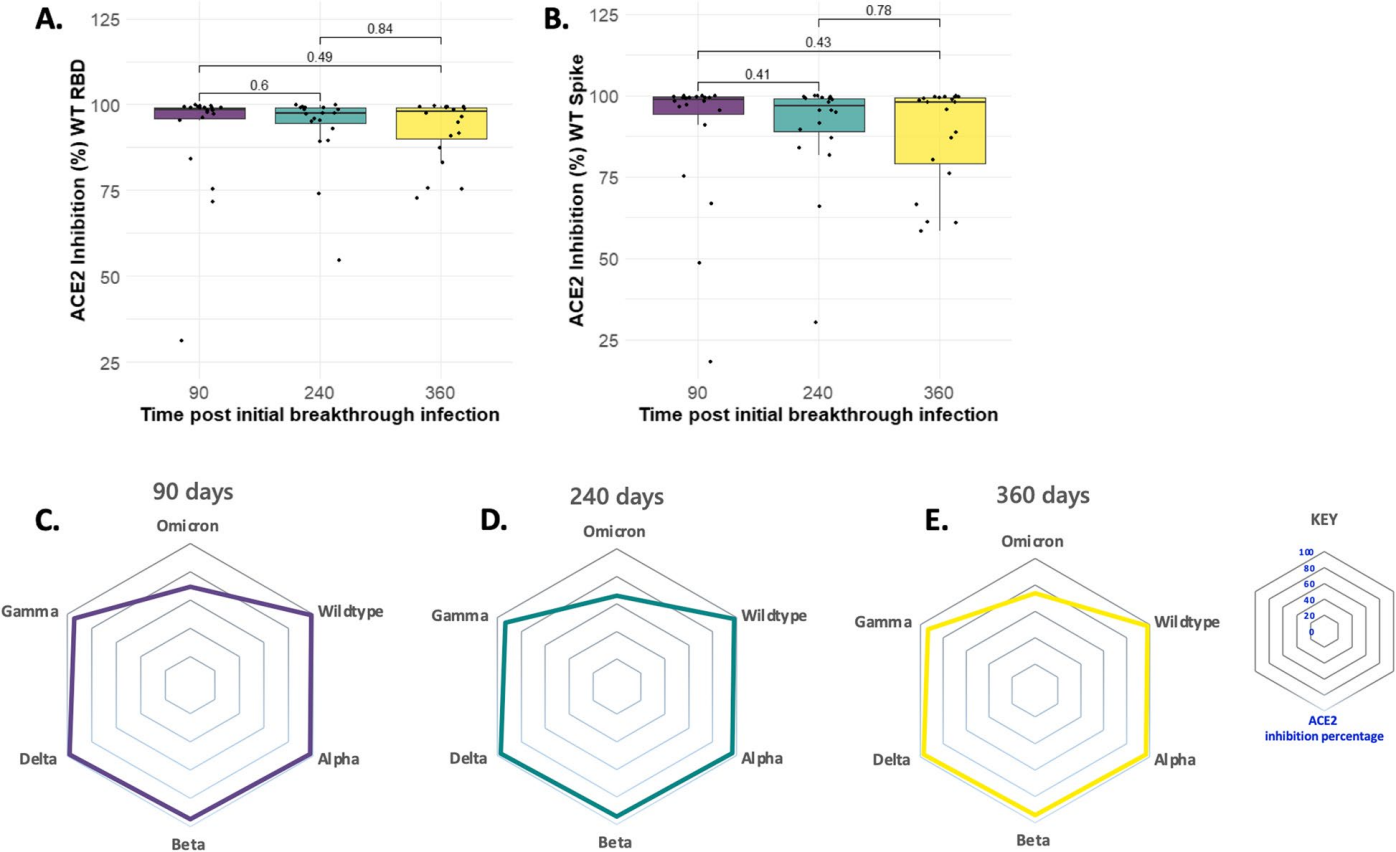
Antibody neutralisation capacity post SARS-CoV-2 breakthrough infection overtime. Box plots showing magnitude of neutralising antibodies against (**a**) anti-RBD ACE2 inhibiting antibodies and (**b**) anti-Spike ACE2 inhibiting antibodies. The horizontal bars represent the median and interquartile range (IQR). Statistics were calculated using Wilcoxon rank sum test and *p* value adjusted for multiple comparisons using Bonferroni multiple comparisons test. A *p*<0.05 was regarded as statistically significant. Radar charts in (**c**-**e**) represent median ACE2 inhibitory percentages against VOCs (Wild type, Alpha, Beta, Delta, Gamma, and Omicron) calculated at 90 days, 240 days, and 360 days post initial SARS-CoV-2 breakthrough infection

To ascertain the neutralisation breadth of SARS-CoV-2 binding antibodies, we quantified ACE2 inhibiting Spike and RBD antibodies in the sera obtained at 90, 240 and 360 days post-initial breakthrough infection. Anti-ACE2 inhibition antibodies were used as a surrogate of neutralisation capacity. First, we compared the neutralisation capacity of serum anti-RBD and anti-Spike-specific antibodies using ancestral antigens. We found that there was no significant difference in the neutralisation potency across the three time points (Fig. 4a-b). Second, we compared the neutralisation breadth of anti-Spike-specific IgG antibodies against Wild-type, Alpha, Beta, Gamma, Delta, and Omicron (B.1.1.529). We found that the neutralisation breadth was maintained for at least 360 days after initial breakthrough infection (Fig. 4c-e). Additionally, ACE-2 inhibition percentage against Delta, Gamma, Beta, Alpha, and Wild-type was above 90% during the study follow-up period but was less than 80% against Omicron (Fig. 4c-e). Collectively, this data indicate that hybrid immunity generated in the context of an adenovirus-based COVID-19 vaccine and breakthrough infection is not only broadly cross-neutralising but also maintained for at least 360 days.

## Discussion

Hybrid immunity is associated with broadly cross-neutralising antibodies [9–11] and provides better protection against COVID-19 than vaccination or prior natural infection alone [7, 8]. However, it is still unclear how long antibodies from hybrid immunity last, particularly in adults vaccinated with an adenovirus-based COVID-19 vaccine. In this study, we demonstrate that adults vaccinated with an adenovirus-based vaccine who had SARS-CoV-2 infection following vaccination can mount durable hybrid immunity. This hybrid immunity is characterised by high levels of serum cross-reactive neutralising anti-Spike IgG antibodies, which can last for at least 360 days. This finding is consistent with a meta-analysis showing that the effectiveness of hybrid immunity against severe COVID-19 was 97·4% at 12 months with primary series vaccination [8].

In Malawi, there appeared to be a seasonal trend in the COVID-19 pandemic, with a rise in cases occurring approximately every six months in January and July from 2020 to 2022 [21]. This pattern is consistent with the kinetics of anti-nucleocapsid IgG antibodies observed in this study, which were associated with six monthly peaks, suggestive of SARS-CoV-2 re-exposure events. Interestingly, these potential re-exposure events were accompanied by a concurrent increase in serum anti-Spike and anti-RBD IgG antibodies, resulting in the maintenance of binding antibodies above the reported putative protective levels [19]. Although hybrid immunity does not provide sterilising immunity, it significantly reduces the risk of severe COVID-19 [8]. In agreement with this, despite the multiple SARS-CoV-2 re-exposure events, none of the participants experienced severe COVID-19 or reported PCR-confirmed symptomatic COVID-19 during the one-year study follow-up.

Despite the considerable strength of the study, which includes a longer follow-up period, consistent monthly sampling, and robust serological assays, it has some limitations. First, it is possible that the changes in anti-nucleocapsid IgG antibodies, which are sometimes interpreted as evidence of re-exposure to SARS-CoV-2 [20], may not necessarily be caused by re-infection but by other factors including infection with human coronaviruses of the common cold. Moreover, rises in anti-nucleocapsid IgG antibodies following SARS-CoV-2 infection are less robust in vaccinated individuals [22], likely due to reduced viral replication. Hence, there is a potential for underestimating SARS-CoV-2 re-infection events. However, since previous studies have shown that serum anti-nucleocapsid IgG antibodies tend to wane rapidly [23, 24], a significant increase in their titres is more likely because of SARS-CoV-2 re-infection. Second, there is a possibility of recall bias in reported clinical events during the follow-up period, such as symptomatic COVID-19. However, since the participants were followed monthly, it is unlikely that they would have difficulty remembering whether they had been hospitalised or had experienced severe COVID-19 in the previous 30 days. Third, due to the small sample size, we could not further segregate the antibody responses based on vaccination doses, vaccine type, timing of vaccination/infection, infecting variant, or number of re-infections. This highlights the complexity of the current COVID-19 immune landscape and indicates that hybrid immunity is very heterogeneous.

## Conclusions

Our study provides serological evidence that indicates that hybrid immunity in adults who have received an adenovirus-based COVID-19 vaccine can last for at least a year. The study highlights the durability of cross-reactive neutralising IgG antibodies. It also suggests that re-exposure from non-severe SARS-CoV-2 infections likely plays a role in maintaining hybrid immunity in previously vaccinated adults.

## Data Availability

All data generated or analysed during this study are available upon reasonable request from the corresponding author.

## Abbreviations

COVID-19: Coronavirus disease 2019
SARS-CoV-2: Severe Acute Respiratory Syndrome Coronavirus 2
RBD: Receptor Binding Domain
IgG: Immunoglobulin G
ACE-2: Angiotensin-converting enzyme 2
mRNA: Messenger Ribonucleic Acid
